# Accounting for population structure in genomic predictions of *Eucalyptus globulus*

**DOI:** 10.1093/g3journal/jkac180

**Published:** 2022-08-03

**Authors:** Andrew N Callister, Matias Bermann, Stephen Elms, Ben P Bradshaw, Daniela Lourenco, Jeremy T Brawner

**Affiliations:** Treehouse Forest Research LLC, Check, VA 24072, USA; Department of Animal and Dairy Science, University of Georgia, Athens, GA 30602, USA; HVP Plantations, Churchill, VIC 3842, Australia; Australian Bluegum Plantations, Albany, WA 6330, Australia; Department of Animal and Dairy Science, University of Georgia, Athens, GA 30602, USA; Department of Plant Pathology, University of Florida, Gainesville, FL 32611, USA

**Keywords:** breeding value accuracy, cross-validation, forest tree breeding, genetic groups, genomic selection, LR method, metafounders, Myrtaceae, single-step GBLUP, MPP, Multiparental Populations, Multiparent Advanced Generation Inter-Cross (MAGIC)

## Abstract

Genetic groups have been widely adopted in tree breeding to account for provenance effects within pedigree-derived relationship matrices. However, provenances or genetic groups have not yet been incorporated into single-step genomic BLUP (“HBLUP”) analyses of tree populations. To quantify the impact of accounting for population structure in *Eucalyptus globulus*, we used HBLUP to compare breeding value predictions from models excluding base population effects and models including either fixed genetic groups or the marker-derived proxies, also known as metafounders. Full-sib families from 2 separate breeding populations were evaluated across 13 sites in the “Green Triangle” region of Australia. Gamma matrices (Γ) describing similarities among metafounders reflected the geographic distribution of populations and the origins of 2 land races were identified. Diagonal elements of Γ provided population diversity or allelic covariation estimates between 0.24 and 0.56. Genetic group solutions were strongly correlated with metafounder solutions across models and metafounder effects influenced the genetic solutions of base population parents. The accuracy, stability, dispersion, and bias of model solutions were compared using the linear regression method. Addition of genomic information increased accuracy from 0.41 to 0.47 and stability from 0.68 to 0.71, while increasing bias slightly. Dispersion was within 0.10 of the ideal value (1.0) for all models. Although inclusion of metafounders did not strongly affect accuracy or stability and had mixed effects on bias, we nevertheless recommend the incorporation of metafounders in prediction models to represent the hierarchical genetic population structure of recently domesticated populations.

## Introduction

Provenance variation in growth and commercial value is usually characterized at an early stage in the domestication of forest trees and can play an important role in subsequent generations of breeding (White *et al.* 2007). Forest geneticists have widely adopted genetic groups to account for population differences in linear mixed models (LMM) (e.g. McRae *et al.* 2004; Brawner *et al.* 2010; Callister *et al.* 2011; Klápště *et al.* 2019), typically as unknown parent groups (UPGs) that were developed to account for missing parents in livestock populations (Quaas 1988; Westell *et al.* 1988). This approach treats provenances as fixed effects, and it results in more conservative estimates of heritability relative to treating founders as unrelated individuals (Callister *et al.* 2021).

Widespread adoption of genomics in breeding programs has prompted the development of the single-step genomic BLUP method, which merges empirical relationship estimates from a genotyped subset (**G** matrix) with the pedigree-derived relationship coefficients (**A**) for the entire population (Legarra *et al.* 2009; Aguilar *et al.* 2010; Christensen and Lund 2010). The resulting inverse relationship matrix (**H**^−1^) is then used in place of **A**^−1^ in LMM to produce (genomic) estimated breeding values ((G)EBV). Although there have been a number of studies applying this “HBLUP” approach to forest tree populations (e.g. Ratcliffe *et al.* 2017; Klápště *et al.* 2018; Thavamanikumar *et al.* 2020; Ukrainetz and Mansfield 2020; Callister *et al.* 2021; Jurcic *et al.* 2021), none have yet considered multiple base populations such as provenances.

UPGs have been incorporated into livestock HBLUP analyses through various modifications of **H**^−1^ that extend the method of Quaas (1988) and Westell *et al.* (1988) (Misztal *et al.* 2013; Tsuruta *et al.* 2019; Masuda *et al.* 2021). A similar approach is to constitute genetic groups as “metafounders” (MFs), which are pseudo-individuals acting as proxies for UPGs in base populations (Legarra *et al.* 2015; Masuda *et al.* 2022). Genetic diversity within, and relationships among MFs, are computed using the genotypes of their descendants. MF models have performed favorably in both simulated and actual livestock populations (Bradford *et al.* 2019; Kudinov *et al.* 2020; Macedo, Christensen, *et al.* 2020; Masuda *et al.* 2021). The MF approach is of particular interest for representing provenances of forest populations because it may estimate biologically meaningful relationships among geographically or genetically defined races that arise from evolutionary processes. MFs may also reveal cryptic relationships between these races and infusions from external populations.

Predictions from quantitative genetic models can be assessed against 3 important parameters: accuracy, bias, and dispersion. Accuracy is a measure of the reliability of EBVs and is used to predict the response to selection (Bijma 2012). Bias is the difference between means of EBVs and true breeding values (TBVs), whereas dispersion is calculated as the slope of the regression of TBVs on EBVs and compares the scale or variation of EBVs against TBVs. Dispersion values less than 1 indicate over-dispersion and inflation of EBV relative to TBV, whereas values greater than 1 indicate under-dispersion of EBV. Bias and dispersion influence expected genetic gain, with potentially significant impacts on selection decisions (Macedo, Reverter, *et al.* 2020). Legarra and Reverter (2018) introduced the linear regression (LR) method to evaluate accuracy, bias, and dispersion using a cross-validation approach.

*Eucalyptus globulus* Labill. is a commercially important plantation species in Mediterranean climates around the world, and it is particularly favored for pulpwood production. Its native range in Southeast Australia has been classified into 13 races and 20 subraces using morphological and molecular markers (Dutkowski and Potts 1999; Potts *et al.* 2004). Landraces have also developed where *E. globulus* has been naturalized in areas such as California, Portugal, Spain, and Chile. Genetic associations among races have been shown to follow geographical patterns (Costa *et al.* 2017), with Portuguese and Californian landraces shown to be most closely related to the east Tasmanian races (Freeman *et al.* 2007; Costa *et al.* 2017; Yost *et al.* 2021).

Callister *et al.* (2021) used HBLUP to conduct a joint analysis of 2 disconnected *E. globulus* breeding populations that were linked using marker-derived pedigree. Relationships within the resulting **H** matrix tended to be significantly positive among population founders from the same population, while across-race relationships among founders ranged from significantly positive to significantly negative. The authors suggested that true average relationships within races were probably substantially larger than those estimated in **H** and that accommodating ancestral race effects in HBLUP should be prioritized. The goals for the present study extend from this recent work (Callister *et al.* 2021) using a subset of their data. Our goals were to (1) fit models including MF and examine relationships corresponding to race, landrace, and plus tree (infusion) populations and (2) use the LR approach (Legarra and Reverter 2018) to evaluate goodness of fit for genetic models with and without genomic relationship information, with genetic groups excluded or incorporated as UPGs, or as MFs.

## Materials and methods

### Experimental populations

**Fig. 1.**
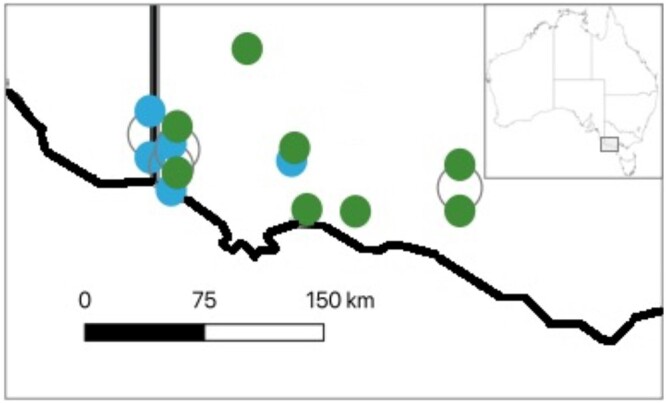
Location of 5 progeny trials from the EG1 program (blue symbols) and 8 progeny trials from the EG2 program (green symbols) used for this study. Inset provides context.

This study used a subset of the *E. globulus* phenotype data analyzed by Callister *et al.* (2021). Full-sib family data for 13 progeny tests in the “Green Triangle” region of Australia were provided by 2 separate multigenerational commercial tree improvement programs, EG1 (Australian Bluegum Plantations) and EG2 (HVP Plantations) (Fig. 1).

Pedigree founders or base population parents were allocated to 14 genetic groups, which consisted of 9 races (following Dutkowski and Potts 1999), 2 landraces (Portugal and California), and 3 plus tree populations (see Supplementary Table 1). Plus tree populations 1 and 2 corresponded to external sources of improved populations from which phenotypic selections were made. Miscellaneous unpedigreed individuals from unknown origins or external sources that lacked sufficient representation to constitute their own groups were allocated to plus tree population 3. The pedigree contained 35,533 progeny (35,247 of which were phenotyped), 368 parents of progeny, 175 progenitors of those parents, and 172 other seed orchard individuals that were both related and unrelated to the tested progeny (see Supplementary Table 2). Progeny represented 650 full-sib families from EG1 and 592 full-sib families from EG2.

HBLUP was necessary to analyze joint-program data due to the inadequacy of inter-program pedigree relationships. Callister *et al.* (2021) reported 2,071 inter-program relationship coefficients greater than 0.1, 428 greater than 0.2, and 70 greater than 0.3 in a genomic relationship matrix (**G**) using a subset of the genotyped individuals from the present analysis, which provides ample connectivity for joint HBLUP models.

Field trials were primarily established in randomized incomplete-block designs. Four to eight replications of each family were established in contiguous row-plots of 4–5 trees. One EG1 trial was established as a single-tree plot design. Incomplete blocks were arranged into replicates that were generally contiguous, enabling the resolution of spatial trends within each trial.

### Stem volume calculation

Diameter at breast height (DBH) for each tree was measured with a tape at 3 years (4 sites), 4 years (3 sites), 5 years (5 sites), or 8 years (1 site) after planting. Tree height (HT) was measured with a hypsometer for each tree in the 5 EG1 trials, and for 19% of trees in EG2 trials. In these cases, unmeasured HT data were predicted from DBH-HT relationships established among measured trees for each trial. Stem volume (VOL) was calculated for each tree using DBH, HT (measured or predicted), and the volume function provided by each breeding program. DBH of all stems forking below breast height was measured in ten trials and in these cases, VOL was aggregated to the tree level before analysis.

### Genotyping

Leaves were sampled from 148 parents and 89 others in the EG1 program and from 80 parents, 958 progeny, and 32 others in the EG2 program, where “others” refers to individuals that are not parents or tested progeny. DNA extraction and genotyping were conducted by Gondwana Genomics Pty Ltd, Canberra (Thavamanikumar *et al.* 2020). The *E. globulus* marker panel consisted of 2,568 single-nucleotide polymorphism (SNP) and small biallelic insertion/deletion (INDEL) markers within candidate genes identified in previous studies (Southerton *et al.* 2011). Missing values were imputed using LinkImpute (Money *et al.* 2015). Pedigree errors were identified and corrected within the genotyped population using the ASRgenomics package (Gezan *et al.* 2021) in R version 4.1.0 (R Core Team 2021).

### Statistical analyses

We conducted 2 sets of analyses, each including various models to compare methods of incorporating genetic groups into analyses. A set of *single-population* analyses was completed using the EG2 population to contrast pedigree-based BLUP (ABLUP) with HBLUP models. A set of *joint-population* HBLUP analyses were completed using information from both programs, with a joint **H** for unification.

#### Calculation of Γ

The relationship matrix across MF, Γ, is a function of genetic similarity across populations following Γ_*ij*_ = 8*cov*(*p_i_*, *p_j_*), where *p_i_* represents allele frequencies in population *i* (Garcia-Baccino *et al.* 2017). We calculated Γ using the gammaf90 program of the BLUPF90 software suite (Misztal *et al.* 2014), which estimates allele frequencies, and thus $\hat{\Gamma }$_*ij*_ = 8*cov*($\hat{p}$_*i*_,$\hat{p}$_*j*_) using the GLS method of Garcia-Baccino *et al.* (2017). The Γ used for *single population* analyses was calculated across 9 genetic groups represented in the EG2 program (8 races and 1 landrace; see Supplementary Table 1). Γ for the *joint population* analyses was calculated across all 14 genetic groups.

#### Relationship matrix calculation

The Γ-augmented inverse of the pedigree relationship matrix was calculated as:

(#1) $$A_{\Gamma }^{-1}=\left[\begin{array}{ccc} A_{\Gamma }^{11} & A_{\Gamma }^{12} & A_{\Gamma }^{1m}\\ A_{\Gamma }^{21} & A_{\Gamma }^{22} & A_{\Gamma }^{2m}\\ A_{\Gamma }^{m1} & A_{\Gamma }^{m2} & A_{\Gamma }^{mm}+\Gamma^{-1} \end{array}\right],$$

where the superscript “1” represents ungenotyped individuals, “2” represents genotyped individuals, and “*m*” represents MF.

Without consideration of genetic groups, the inverse of **H** was calculated as:

(#2) $$H^{-1}=\;A^{-1}+\;\left[\begin{array}{cc} 0 & 0\\ 0 & G^{-1}-A_{22}^{-1} \end{array}\right].$$

Following Christensen *et al.* (2012) and Aguilar *et al.* (2010), **G** was blended as 0.95**G**_scaled_ + 0.05**A**_22_, where **G**_scaled_ was a rescaled version of **G**_obs_, originally computed from observed allele frequencies by the first method of VanRaden (2008). For HBLUP with MFs, the Γ-augmented $H^{-1}\;(H_{\Gamma }^{-1})$ was calculated as:

(#3) $$H_{\Gamma }^{-1}=\;A_{\Gamma }^{-1}+\;\left[\begin{array}{ccc} 0 & 0 & 0\\ 0 & G_{05}^{-1}-A_{\Gamma 22}^{-1} & 0\\ 0 & 0 & 0 \end{array}\right],$$

where $A_{\Gamma 22}^{-1}$ is the inverse of Γ-augmented pedigree relationship matrix ($A_{\Gamma }$) for genotyped individuals and **G**_05_ was calculated in the same manner as **G**, except that the initial computation of the relationship matrix was based on 0.5 allele frequencies (Legarra *et al.* 2015).

To examine relationships among genotyped individuals of the *single program*, $A_{22}$ and $A_{\Gamma 22}$ were calculated from versions of $A^{-1}$ and $A_{\Gamma }^{-1}$ produced with a subset of the pedigree. $H_{\Gamma }^{-1}$ was also produced with a *single program* pedigree subset small enough for convenient inversion and inspection.

#### Model fitting

Stem volume data were first analyzed at the individual site level using ASReml-R version 4 (Butler *et al.* 2018) in R version 4.1.0 to adjust the data for any trial design effects (incomplete block, row, column, or plot) and spatial trend (AR1xAR1), represented as random effects. The adjusted data were then standardized by trial to a mean of zero and phenotypic standard deviation of 1.

All cross-site models were fitted using BLUPF90 programs and the general LMM framework:

(#4) $$y=\mathrm{Xb}+Z_{1}a\;+Z_{2}f+e,$$

where **y** is the vector of phenotypic values, **b** is a vector of fixed effects for sites, **a** is the vector of random additive genetic effects with *E*(**a**) = 0, **f** is a vector of random family-specific effects with *E*(**f**) = 0 and var(**f**) = ${I\sigma }_{f}^{2}$, and **e** is the vector of residual effects with *E*(**e**) = 0 and var(**e**) =$\;{I\sigma }_{e}^{2}$. **X**, **Z_1_**, and **Z_2_** are incidence matrices relating phenotypic records in **y** to effects in vectors **b**, **a**, and **f**.

Five models were fitted to the *single population* which differed only in the specification of var(**a**). Three pedigree models were fitted: without groups (ABLUP), with fixed genetic groups (ABLUP_UPG), and with MFs (ABLUP_MF). For ABLUP, var(**a**) = **A**$\sigma_{a}^{2}$. For ABLUP_UPG, var(**a**) = ${A^{*}\sigma }_{a}^{2}$, where $A^{*}$ is adjusted to include fixed group effects (Quaas 1988; Westell *et al.* 1988). For ABLUP_MF, var(**a**) = ${A_{\Gamma }\sigma }_{a}^{2}$, where $A_{\Gamma }$ was calculated using Equation 1. Two HBLUP models were fitted to the *single population* and *joint population.* Model “HBLUP” without groups assumes var(**a**) = **H**$\sigma_{a}^{2}$, whereas HBLUP models with MFs (HBLUP_MF) were fitted assuming var(**a**) = ${H_{\Gamma }\sigma }_{a}^{2}$.

Variances were specified for all random effects at values estimated from ABLUP for the *single population* analyses and from HBLUP for the *joint population* analyses. Additive variance for MF models was divided by a scalar $k=1+\;\overset{-}{\mathrm{diag}(\Gamma )}/2-\;\overset{-}{\Gamma }$ (Legarra *et al.* 2015), which was 1.010 for the *single population* and 0.987 for the *joint population*.

#### Evaluation of model performance

Model performance was evaluated using the LR method (Legarra and Reverter 2018). This approach involves removing phenotypes from a focal group to create a partial dataset, which is analyzed with the same model specification as the complete phenotypic dataset. Solutions from analysis of the whole dataset are then regressed against solutions obtained from the partial dataset to provide model validation statistics. We formed focal groups consisting of 5,400 progeny from the *single population* (all progeny of 20 parents representing a diversity of origins) and 8,367 progeny from the *joint population* (all progeny of 60 parents representing a diversity of origins and both programs). Legarra and Reverter (2018) showed that regressions may be formed among solutions for either the retained individuals or the focal individuals. We used the focal group as our goal was to determine the best model for predicting breeding values of unphenotyped individuals in the pedigree. For each model specification, prediction accuracy (ACC) was calculated as $\sqrt{\frac{\mathrm{cov}({\hat{a}}_{w},{\hat{a}}_{p})}{(1-\overset{-}{F}){\hat{\sigma }}_{a}^{2}}}$ and stability (STAB) as the correlation $\frac{\mathrm{cov}({\hat{a}}_{w},{\hat{a}}_{p})}{\sqrt{v\mathrm{ar}({\hat{a}}_{w})\mathrm{var}({\hat{a}}_{p})}}$, where $\mathrm{cov}({\hat{a}}_{w},{\hat{a}}_{p})$ is the covariance between focal group solutions from the whole and partial datasets, $\overset{-}{F}$ is average inbreeding coefficient of the focal group calculated from the **H** matrix without groups using the INBUPGF90 program (Misztal *et al.* 2014), and ${\hat{\sigma }}_{a}^{2}$ is the REML estimate of the additive genetic variance. Dispersion (DISP) was calculated as the regression slope: $\mathrm{cov}({\hat{a}}_{w},{\hat{a}}_{p})/\mathrm{var}({\hat{a}}_{p})$. BIAS was calculated as $\overset{-}{\hat{a_{p}}}-\overset{-}{\hat{a_{w}}}$, where $\overset{-}{\hat{a_{p}}}$ and $\overset{-}{\hat{a_{w}}}$ are mean focal group solutions from analysis of the partial and whole datasets, respectively.

## Results

### Relationship matrices

**Fig. 2.**
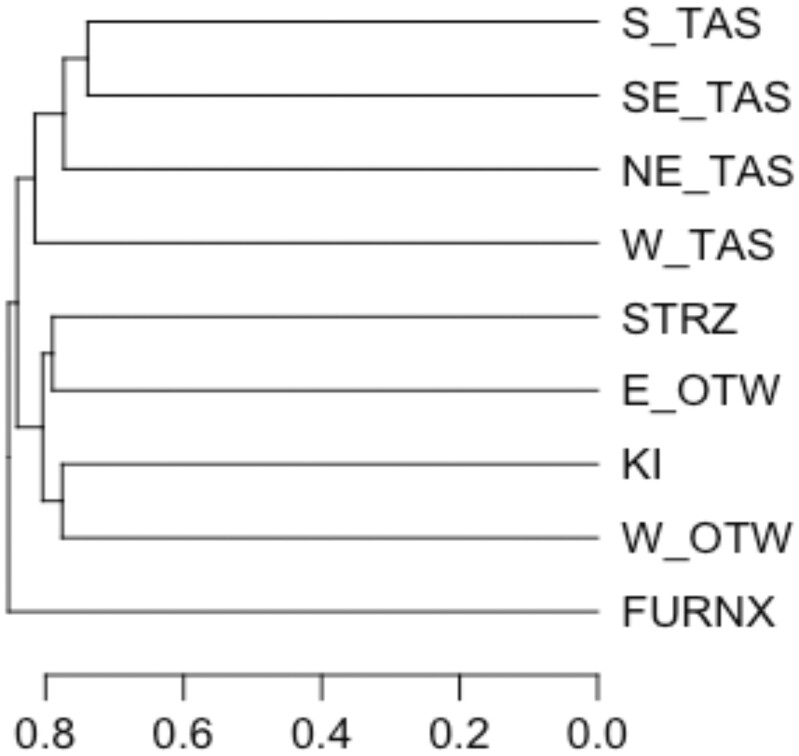
UPGMA plot of similarity among *E. globulus* races based on Γ. See Supplementary Table 1 for key to race names.

The Γ estimate for the *joint population* was:
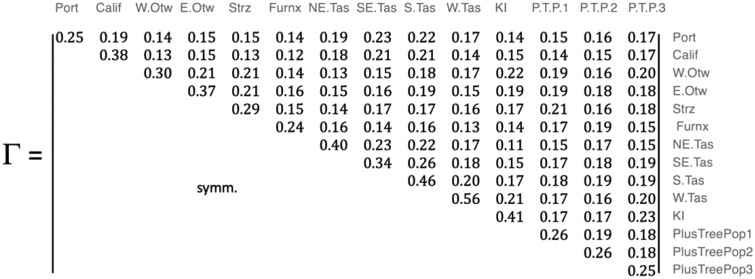


Diagonal elements of Γ ranged from 0.24 to 0.56 with a mean of 0.34, which corresponds to a mean inbreeding coefficient of −0.64 within MF. Plus-Tree Populations were more genetically diverse than the average MF, with $\Gamma_{ii}$ estimates lower than the average of the diagonal elements. Off-diagonal elements of Γ provide estimates of relatedness among populations that are derived from correlations of allele frequencies. Γ showed there are closer affinities amongst East Otways, West Otways, and Strzelecki races from the mainland of Australia, and King Island population was most similar to the West Otways population. Closer affinities between the 3 east-coast Tasmanian races were evident in Γ, with the greatest between-population relationship coefficient found between South-eastern Tasmania and Southern Tasmania ($\Gamma_{ij}$ = 0.26). A UPGMA phylogenetic tree representing the among-race relationships in Γ demonstrates these 2 clusters, while associating Western Tasmania loosely with east Tasmanian races and showing that Furneaux is the most distantly related race (Fig. 2). The Portuguese and Californian landraces were most closely related to South-eastern Tasmania and Southern Tasmania in Γ. Plus-Tree Population 1 was most closely related to the Strzelecki race ($\Gamma_{ij}$ = 0.21) and Plus-Tree Population 2 was not strongly associated with any particular race in Γ, while Plus-Tree Population 3 was most closely aligned with King Island ($\Gamma_{ij}$ = 0.23).

In contrast with the HBLUP method, which modifies the genomic relationship matrix to be compatible with **A**, the HBLUP_MF method modifies **A** to be compatible with $G_{05}$. Table 1 shows correlations among elements of the $A_{22}$, $A_{\Gamma 22}$, $G_{\mathrm{obs}}$, and $G_{05}$ matrices in the EG2 population. Correlations between diagonal and off-diagonal elements of $A_{22}$ and $G_{\mathrm{obs}}$ were 0.08 and 0.85, respectively (Table 1). Inclusion of MF relationships in **A** increased the similarity of pedigree and genomic relationship matrices. The correlations between diagonal and off-diagonal elements of $A_{\Gamma 22}$ and $G_{05}$ were 0.81 and 0.87, respectively (Table 1).

**Table 1.** Correlations among diagonal (upper triangle) and off-diagonal (lower triangle) elements of $A_{22}$, $A_{\Gamma 22}$, $G_{\mathrm{obs}}$, and $G_{05}$.

	$A_{22}$	$A_{\Gamma 22}$	$G_{\mathrm{obs}}$	$G_{05}$
$A_{22}$	1	0.62	0.08	0.14
$A_{\Gamma 22}$	0.96	1	0.89	0.81
$G_{\mathrm{obs}}$	0.85	0.30	1	0.30
$G_{05}$	0.77	0.87	0.91	1

As expected, $G_{\mathrm{obs}}$, calculated with observed allele frequencies, displayed a similar mean to $A_{22}$, but with greater variation (Table 2). $A_{\Gamma 22}\;$had slightly higher diagonal values than $A_{22}$, as inbreeding values were translated from Γ throughout the pedigree and each founder’s inbreeding value in $A_{\Gamma }$ incorporates half of $\Gamma_{ii}$ from its respective MF. The mean off-diagonal element of $A_{\Gamma 22}$ was substantially greater than that of $A_{22}$ (Table 2), as relationships within and among MFs in Γ were distributed throughout $A_{\Gamma }$. For example, founders with a common MF that were unrelated in **A** were related in $A_{\Gamma }$ by $\Gamma_{ii}$, the diagonal value corresponding to their common MF. Similarly, founders from different MF that were unrelated in **A** were related in $A_{\Gamma }$ by $\Gamma_{ij}$, the off-diagonal value corresponding to the relationship between their respective MFs. Relationships at the founder level therefore influence relationships among all parents and progeny in $A_{\Gamma }$. Values in $G_{05}$ were considerably greater than all other relationship matrices among genotyped individuals (Table 2). Diagonal elements of the $H_{\Gamma }$ matrix representing *single program* parents and progenitors were consistently greater than the corresponding elements of A with UPG, ranging from 1.10 to 1.67, with a mean of 1.41.

**Table 2.** Mean, minimum, and maximum element values of $A_{22}$, $A_{\Gamma 22}$, $G_{\mathrm{obs}}$, and $G_{05}$ on the diagonal and off-diagonal.

Element	Matrix	Mean	Min	Max
Diagonal	$A_{22}$	1.00	1.00	1.25
	$A_{\Gamma 22}$	1.07	1.04	1.35
	$G_{\mathrm{obs}}$	1.04	0.78	1.38
	$G_{05}$	1.34	1.13	1.71
Off-diagonal	$A_{22}$	0.03	0.00	0.75
	$A_{\Gamma 22}$	0.19	0.08	0.92
	$G_{\mathrm{obs}}$	0.00	−0.24	0.85
	$G_{05}$	0.61	0.40	1.22

### Model results

Additive variance estimates were similar between the *single population* and *joint population* (around 0.17; Table 3), corresponding to narrow-sense heritability estimates of 0.168 for the *single population* and 0.161 for the *joint population* (Table 3). Family-specific variances were also similar between the *single population* and *joint population* (around 0.04; Table 3). Genetic group solutions from ABLUP_UPG were strongly correlated with MF solutions from ABLUP_MF and HBLUP_MF in the *single population* (*r* 0.97 and 0.96, respectively).

**Table 3.** Variance components from *single population* ABLUP and *joint population* HBLUP.

Var. comp.^*a*^	*Single pop.*	*Joint pop.*
${\hat{\sigma }}_{a}^{2}$	0.171	0.164
${\hat{\sigma }}_{f}^{2}$	0.043	0.042
$\bar{{\hat{\sigma }}_{e}^{2}}$ ± SD(${\hat{\sigma }}_{e}^{2}$)^*b*^	0.805 ± 0.040	0.812 ± 0.038
${\hat{h}}_{\mathrm{Xsite}}^{2}$	0.168	0.161

a ${\hat{\sigma }}_{a}^{2}\;$ is the additive variance, ${\hat{\sigma }}_{f}^{2}$ is the family-specific variance, ${\hat{\sigma }}_{e}^{2}$ is the site error variance, and ${\hat{h}}_{\mathrm{Xsite}}^{2}$ is the cross-site narrow-sense heritability.

b Mean and standard deviation of 8 *single population* site error estimates and 13 *joint population* site error estimates.

**Fig. 3.**
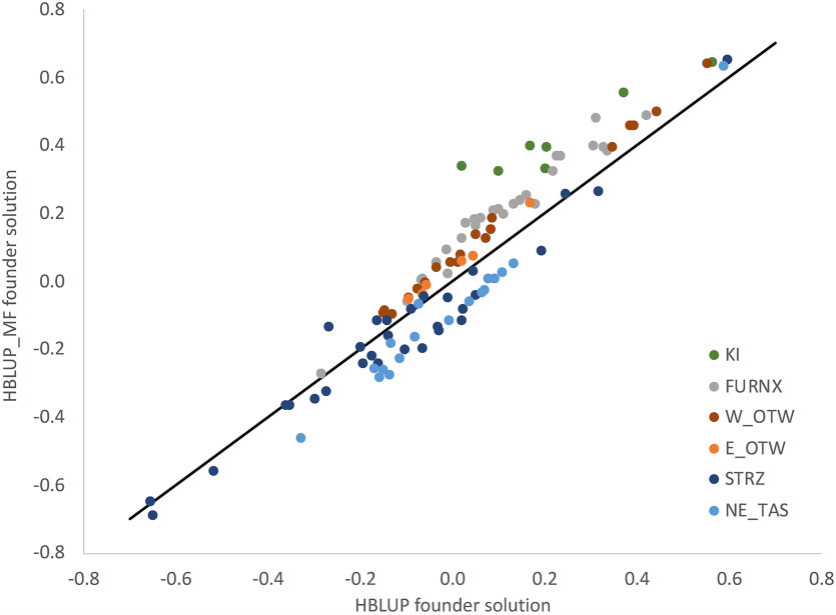
Relationships between *joint population* HBLUP_MF and HBLUP solutions for founders from 6 races selected for demonstration purposes. See Supplementary Table 1 for key to race names.

The inclusion of MF influenced (G)EBVs of individuals from HBLUP_MF analyses. This is demonstrated in Fig. 3, where founders’ HBLUP_MF solutions are both higher and lower than their HBLUP solutions, depending on genetic group assignment.

### Evaluation of models using the LR method

Accuracy of the *single population* focal group was increased by addition of genomic information, from 0.41 for ABLUP to 0.47 for HBLUP (Table 4). Inclusion of genetic groups also increased ACC of pedigree-based solutions, to 0.50 with UPG or 0.46 with MF (Table 4). The ACC of HBLUP solutions was slightly lower with the inclusion of MF for both the *single population* and *joint population*, reduced by 0.02 and 0.01, respectively.

**Table 4.** Results of linear regression validation: accuracy, stability, dispersion, and bias, expressed in dm^3^ units with genetic standard deviation units in parentheses.

Population	Model	Acc	Stab	Disp	Bias (${\mathrm{SD}}_{\hat{a}}$)
Single program	ABLUP	0.41	0.68	1.09	0.038 (0.14)
	ABLUP_UPG	0.50	0.76	0.97	0.046 (0.17)
	ABLUP_MF	0.46	0.77	1.10	0.048 (0.18)
	HBLUP	0.47	0.71	0.95	0.066 (0.25)
	HBLUP_MF	0.45	0.72	0.96	−0.006 (−0.02)
Joint program	HBLUP	0.45	0.66	0.90	0.034 (0.13)
	HBLUP_MF	0.44	0.69	0.91	0.067 (0.27)

Stability was increased in the *single population* from 0.68 for ABLUP to 0.71 for HBLUP, 0.76 for ABLUP_UPG, and 0.77 for ABLUP_MF (Table 4). Stability was increased by addition of MF to HBLUP, by 0.01 for *single population* and 0.03 for *joint population.*

Dispersion was generally acceptable as estimates were within ±0.1 of 1.0 (Table 4). Nevertheless, ABLUP and ABLUP_MF models were over-dispersed, and ABLUP_UPG produced the DISP value closest to 1.0. DISP was similar between HBLUP models with and without MF (Table 4).

Bias was similar among ABLUP models regardless of how genetic groups were treated (Table 4). Inclusion of MF in HBLUP models produced contrasting results. For the *single population*, MF reduced bias from +0.066 to −0.006, whereas for the *joint population*, MF increased bias from +0.034 to +0.067 (Table 4).

## Discussion

Forest tree breeding programs are often founded on diverse sets of parents from wild populations with distinct characteristics that have developed as adaptations to local environments. Inclusion of provenances as genetic groups uses these similarities to improve the estimation of genetic parameters and effects in a range of tree breeding contexts (e.g. Callister *et al.* 2011; Lee *et al.* 2015; Ukrainetz *et al.* 2018). Thoughtful genetic group definitions for unpedigreed individuals can elucidate patterns of gene flow as well as improve predictions (Klápště *et al.* 2019). There is growing interest in the HBLUP approach to integrate genomic, pedigree, and phenotypic information and provide unbiased predictions of genetic merit for tree breeding programs (Callister *et al.* 2021). Accounting for population structure with HBLUP is therefore an important development for tree breeding programs with genotyped subpopulations.

The MF approach is an elegant solution to the challenge of accounting for distinct base populations in HBLUP and it is under extensive investigation in livestock breeding (Masuda *et al.* 2022), where applications include accounting for trends in genetic means of unknown parents across time (e.g. Granado-Tajada *et al.* 2020) and accounting for breeds in crossbred populations (Xiang *et al.* 2017; Kluska *et al.* 2021; Poulsen *et al.* 2022). Our use of MF in a forest tree population now expands the scope of this approach to the plant kingdom, where it may be used to address numerous applications in applied breeding, population genetics, and conservation genetics. The distinct advantage of the MF approach over other genomic methods for population genetic studies is that it provides estimates of relationships among base populations and diversity within groups using the genotype and pedigree of individuals that may be multiple generations removed (Legarra *et al.* 2015).

We have demonstrated that a Γ matrix used to describe MF relationships can be constructed with relatively small founder populations that represent races, landraces, and infused plus tree populations. Diagonal elements of Γ representing our 14 *E. globulus* MFs were substantially smaller than 2/3, which is the expected value if the allele frequencies in the base generation were uniformly distributed (van Grevenhof *et al.* 2019). Estimates of relationships among races in Γ validated previous *E. globulus* population studies that used microsatellite markers (Jones *et al.* 2002; Costa *et al.* 2017) and elucidated relationships between landraces and native races (Freeman *et al.* 2007; Yost *et al.* 2021). Although we expect these patterns across races and landraces to have an empirical basis beyond this particular population, the diagonal elements of Γ appeared to be influenced by sample size of the population. Further estimates of Γ based on different samples would provide welcome validation, particularly from programs with larger *E. globulus* base populations.

As this is the first forestry study with MF, Γ comparisons are restricted to results with livestock populations. Working with cattle, Kluska *et al.* (2021) reported diagonal Γ similar to ours for 4 biological types including subspecies *Bos taurus indicus* and *B. taurus taurus* and 6 combinations among them. Their diagonal Γ values ranged from 0.15 (for the group combining all 4 primary biological types) to 0.65, with a mean of 0.41. Off-diagonal elements of Γ in Kluska *et al.* (2021) tended to be smaller than ours with *E. globulus*, including values of -0.08 and -0.11 between *Bos taurus indicus* and taurine animals from Europe, and from Britain, respectively. Macedo, Christensen, *et al.* (2020) reported diagonal Γ values between 0.52 and 0.96 for diary sheep, with off-diagonal Γ between 0.22 and 0.44. Granado-Tajada *et al.* (2020) also found high diagonal Γ values with diary sheep, ranging up to 1.45, indicating that unknown parents within the 3-year period represented by this certain MF were considerably inbred. Working with red dairy cattle, Kudinov *et al.* (2020) reported Γ with diagonal values from 0.57 to 0.74 and off-diagonals from 0.45 to 0.59, and with chickens, Bermann *et al.* (2021) constructed a Γ with diagonal elements of 0.50 and 0.57 for 2 MF and 0.38 in the off-diagonal.

The values of Γ impact the values of $A_{\Gamma }$, including the subcomponent $A_{\Gamma 22}$. Kudinov *et al.* (2020) created an $A_{\Gamma 22}$ with diagonal and off-diagonal elements on average 0.29 and 0.55 units greater than those in their $A_{22}$, respectively. On the other hand, with considerably smaller values in Γ than those reported by Kudinov *et al.* (2020), our $A_{\Gamma 22}$ was only 0.07 units greater than $A_{22}$ on the diagonal and 0.16 units greater on the off-diagonal, on average (see Table 2). Mean and range of our $G_{05}$ were generally similar to those of Kudinov *et al.* (2020) and Forni *et al.* (2011), who compared various formulations of **G** in a swine population. Our correlations between $G_{05}$ and $A_{\Gamma 22}$ were also similar to those presented by Kudinov *et al.* (2020).

MF effects are treated as random effects, which varies from the typical practice of treating founding populations as fixed effects in forest genetics (White *et al.* 2007). Nevertheless, there are some advantages to treating genetic groups as random effects, and this approach has been considered more seriously by animal geneticists (reviewed by Masuda *et al.* 2022). Theoretical concerns with fixed-effect genetic groups include the lack of inbreeding within groups and the inconsistency of allowing for selection to have altered the means of groups but not their genetic variance (Kennedy 1991). A more pragmatic concern in our experience with tree breeding is that poorly represented genetic groups can be assigned extreme fixed-effect solutions which cause biased EBVs for their relatives throughout the pedigree. Including MF in HBLUP produced changes to (G)EBVs that were similar to including UPG in ABLUP, variously elevating or depressing solutions for founders depending on their race (see Fig. 3). This is an important demonstration of the impact MF are likely to have in applied tree breeding with HBLUP models.

We found that accuracy increased with the addition of genomic information in the *single population*. Breeding value accuracy calculated as a function of prediction error variance has typically been shown to increase from ABLUP to HBLUP in studies of forest trees (e.g. Cappa *et al.* 2017, 2018; Thavamanikumar *et al.* 2020; Callister *et al.* 2021). ACC has been shown to be higher in HBLUP than ABLUP models using the LR method in livestock populations (Cesarani, Biffani, *et al.* 2021; Kluska *et al.* 2021; Sungkhapreecha *et al.* 2021) and by comparing true and estimated (G)EBVs in simulated experiments (Garcia-Baccino *et al.* 2017; Bradford *et al.* 2019; van Grevenhof *et al.* 2019). Addition of MFs to HBLUP models did not improve ACC in our study, which differs from results of simulated populations (Garcia-Baccino *et al.* 2017; Bradford *et al.* 2019; van Grevenhof *et al.* 2019) that showed substantial increases in ACC with the inclusion of MF. Like our result, Kluska *et al.* (2021) found ACC from HBLUP to be lower with MF for most traits, while Bermann *et al.* (2021) showed mixed results across 5 traits. Model specification may affect ACC more for genotyped than ungenotyped individuals (e.g. Ukrainetz and Mansfield 2020) and further comparison of HBLUP models with and without MF is recommended using a larger cohort of genotyped trees and separate LR validations for genotyped and ungenotyped focal groups.

Stability (the correlation between ${\hat{a}}_{p}$ and ${\hat{a}}_{w}$) is also called the “ratio of accuracies” and the expected value is ${\mathrm{acc}}_{p}/{\mathrm{acc}}_{w}$ (Legarra and Reverter 2018; Macedo, Christensen, *et al.* 2020). Our results showed only a small improvement in stability between ABLUP and HBLUP in the *single population*, whereas other studies have demonstrated more marked increases caused by the addition of genomic information (e.g. Cesarani, Biffani, *et al.* 2021; Kluska *et al.* 2021; Sungkhapreecha *et al.* 2021). Including MF improved our HBLUP model stability by 0.01 in the *single population* and 0.03 in the *joint population*, while it had mixed impacts on stability across 4 traits in Kluska *et al.* (2021).

When dispersion is equal to 1.0, the focal group’s (G)EBVs are expressed on the same scale regardless of whether they are phenotyped. Over-dispersion (slope less than 1.0) will cause unphenotyped progeny to be selected in error, whereas under-dispersion (slope greater than 1.0) will cause the best unphenotyped progeny to be overlooked for selection despite their merit. Addition of genomic information has corrected strong over-dispersion found for ABLUP models in actual datasets (e.g. milk yield in Sungkhapreecha *et al.* 2021; direct weaning weight in Kluska *et al.* 2021; all traits in Cesarani, Masuda, *et al.* 2021) and simulated populations (Bradford *et al.* 2019). Other studies (Macedo, Christensen, *et al.* 2020; Cesarani, Biffani, *et al.* 2021) have reported dispersion of various models to be mostly within 10% of the expected value (1.0), which was also our result.

Genetic models ideally have zero bias, so that the mean (G)EBV of unphenotyped trees is correctly predicted. Our bias results were mixed. In the *single population* we found a small positive bias for ABLUP and a larger positive bias for HBLUP, which was effectively eliminated by the inclusion of MF. In contrast, a small positive bias for HBLUP in the *joint population* was doubled by the inclusion of MF, which could be a result of the relatively small number of genotyped individuals contributing to the estimation of Γ. The livestock breeding literature contains a wide range of reports about bias in ABLUP and HBLUP models. Garcia-Baccino *et al.*’s (2017) simulation study found bias to increase markedly with the addition of genomic information and was effectively eliminated with the subsequent inclusion of MF. In Bradford *et al.*’s (2019) simulation study, an incomplete population displayed large positive bias by ABLUP, a much smaller negative bias by HBLUP, and effectively no bias by HBLUP with MF. Cesarani, Biffani, *et al.* (2021) and Sungkhapreecha *et al.* (2021) both found that biases present in ABLUP models were greatly diminished using HBLUP on the same populations. In contrast, Granado-Tajada *et al.* (2020) found that bias in dairy sheep evaluations was greater for HBLUP than ABLUP, although the difference was not statistically significant. Macedo, Christensen, *et al.* (2020) reported the least bias for HBLUP with MF, as did Macedo *et al.* (2022) using a method of Γ construction based on inbreeding trend over time.

## Conclusions

Our results demonstrate that the MF method is well suited to representing multiple founder populations in tree breeding analyses that utilize HBLUP. The Γ matrix can be interpreted to reveal cryptic relationships among UPGs and biologically meaningful associations among provenances or races. Greater confidence in such results will result from validation in future studies with a larger sample of genotyped individuals.

Including MF in HBLUP did not substantially alter model accuracy or dispersion, while it slightly improved stability and presented mixed results for bias in this study. Nevertheless, it is a preferable model on account of its improved approximation of the hierarchical genetic population structure, which we know is present in recently domesticated populations of forest trees. Application of the HBLUP_MF model will therefore provide greater certainty that provenance-level trait variations are being well represented in (G)EBVs of subsequent generations.

## Data availability

The genomic, pedigree, and phenotypic data are available at https://doi.org/10.25387/g3.19487696.

Supplemental material is available at *G3* online.

## Supplementary Material

jkac180_Supplementary_Data
