# Supplementary material for: Accounting for population structure in genomic predictions of Eucalyptus globulus
Source: G3 (Bethesda). 2022 Aug 3;12(9):jkac180. doi: 10.1093/g3journal/jkac180 (PMC9434241; doi:10.1093/g3journal/jkac180)
Supplement: jkac180_Supplementary_Data [file jkac180_supplementary_data.zip › jkac180_Supplementary_Data/Table_SM.A_G3-2022-403610.docx]

**Table SM.A.** Number of founders within *E. globulus* races, land races, and plus tree populations represented in EG1 and EG2 pedigrees

| **Genetic Group** | **Code** | **EG1** | **EG2** | **EG1&2** | **JOINT** |
| --- | --- | --- | --- | --- | --- |
| Western Otways | W_OTW | 8 | 12 | 0 | 20 |
| Eastern Otways | E_OTW | 2 | 5 | 0 | 7 |
| Strzelecki Ranges | STRZ | 9 | 27 | 2 | 38 |
| Furneaux | FURNX | 21 | 6 | 2 | 29 |
| North-eastern Tasmania | NE_TAS | 15 | 3 | 0 | 18 |
| South-eastern Tasmania | SE_TAS | 6 | 8 | 0 | 14 |
| Southern Tasmania | S_TAS | 0 | 3 | 0 | 3 |
| Western Tasmania | W_TAS | 7 | 0 | 0 | 7 |
| King Is. | KI | 1 | 6 | 0 | 7 |
| Portugal | PORT | 17 | 8 | 0 | 25 |
| California | CALIF | 6 | 0 | 0 | 6 |
| Plus-Tree Population 1 | P.T.P.1 | 17 | 0 | 0 | 17 |
| Plus-Tree Population 2 | P.T.P.2 | 12 | 0 | 0 | 12 |
| Plus-Tree Population 3 | P.T.P.3 | 18 | 0 | 0 | 18 |
| **TOTAL** |  | **139** | **78** | **4** | **221** |
