# Supplementary material for: Accounting for population structure in genomic predictions of Eucalyptus globulus
Source: G3 (Bethesda). 2022 Aug 3;12(9):jkac180. doi: 10.1093/g3journal/jkac180 (PMC9434241; doi:10.1093/g3journal/jkac180)
Supplement: jkac180_Supplementary_Data [file jkac180_supplementary_data.zip › jkac180_Supplementary_Data/Table_SM.B_G3-2022-403610.docx]

**Table SM.B.** Summary of pedigree elements for EG1 and EG2 populations. Number of genotyped individuals are in parentheses

|  | **EG1** | **EG2** | **JOINT** |
| --- | --- | --- | --- |
| Progeny | 12,184 (0) | 23,349 (958) | 35,533 (958) |
| Parents of progeny | 263 (148) | 105 (80) | 368 (228) |
| Progenitors of parents | 78 (0) | 97 (0) | 175 (0) |
| Other individuals^a^ | 117 (89) | 55 (32) | 172 (121) |

^a^Unrelated individuals and related individuals that are not parents of tested progeny
